# Exploitation of tumor antigens and construction of immune subtype classifier for mRNA vaccine development in bladder cancer

**DOI:** 10.3389/fimmu.2022.1014638

**Published:** 2022-11-16

**Authors:** Xin Zhang, Yanlong Zhang, Li Zhao, Jiayu Wang, Jiaxing Li, Xi Wang, Min Zhang, Xiaopeng Hu

**Affiliations:** ^1^ Department of Urology, Beijing Chao-Yang Hospital, Capital Medical University, Beijing, China; ^2^ Institute of Urology, Capital Medical University, Beijing, China; ^3^ Institute of Infectious Diseases, Beijing Key Laboratory of Emerging Infectious Diseases, Beijing Ditan Hospital, Capital Medical University, Beijing, China; ^4^ Department of Urology, Beijing Shijitan Hospital, Capital Medical University, Beijing, China; ^5^ School of Pharmacy, Shanxi Medical University, Taiyuan, Shanxi, China; ^6^ Department of Immunology, School of Basic Medical Sciences, Department of Oncology, Capital Medical University, Beijing, China; ^7^ Beijing Institute of Infectious Diseases, Beijing, China; ^8^ Department of Research Ward, Beijing Chao-Yang Hospital, Capital Medical University, Beijing, China

**Keywords:** mRNA vaccine, tumor antigen, bladder cancer, immune subtypes, tumor immune infiltration

## Abstract

**Background:**

Bladder cancer (BLCA) is one of the most prevalent urinary system malignancies, with high mortality and recurrence. The present study aimed to identify potential tumor antigens for mRNA vaccines in BLCA and patient subtypes suitable for different immunotherapy.

**Methods:**

Gene expression profiles, mutation data, methylation data, and corresponding clinical information were obtained from the Cancer Genome Atlas (TCGA), Gene Expression Omnibus (GEO), and ArrayExpress databases. Immunohistochemical staining of microarrays was performed to assess protein expression levels of IGF2BP2 and MMP9. Differential gene analysis, survival analysis, correlation analysis, consensus clustering analysis, and immune cell infiltration analysis were conducted using R software. Finally, the R package “immcluster” was used based on Combat and eXtreme Gradient Boosting algorithms to predict immune clusters of BLCA samples.

**Results:**

Two mutated, amplified, and over-expressed tumor antigens, IGF2BP2 and MMP9, were found to be associated with clinical outcomes and the abundance of antigen-presenting cells (APCs). Subsequently, three immune subtypes (BIS1, BIS2, and BIS3) were defined in the BLCA cohort. BIS3 subtype exhibited an “active” immune phenotype, while BIS1 and BIS2 subtypes have a “suppressive” immune phenotype. Patients in BIS1 and BIS2 had a poor prognosis compared to BIS3. BIS3 had a higher score in checkpoints or immunomodulators (CP) and immunophenoscore (IPS), while BIS1 and BIS2 scored higher in major histocompatibility complex-related molecules (MHC molecules). Meanwhile, BIS2 and BIS3 had a significantly higher tumor mutational burden (TMB) compared to patients with BIS1. Finally, the “immcluster” package was applied to the dataset, which has been shown to accurately predict the immune subtypes of BLCA samples in many cohorts.

**Conclusions:**

IGF2BP2 and MMP9 were potential antigens for developing mRNA vaccines against BLCA. The results in the present study suggested that immunotherapy targeting these two antigens would be suitable for patients falling under the BIS2 subtype. R package “immcluster” could assist in screening suitable BLCA patients for antitumor therapy.

## Introduction

Bladder cancer (BLCA) is one of the most prevalent urinary system malignancies, with more than 83,000 new cases and an estimated 17,200 deaths worldwide in 2020 (1). Risk factors associated with BLCA include advanced age, male, cigarette smoking, chronic inflammation, and occupational exposure containing benzene dyes and factory chemicals, among others (2, 3). BLCA can be generally categorized into non-muscle-invasive bladder cancer (NMIBC) and muscle-invasive bladder cancer (MIBC). In the former, the tumors are isolated in the urothelium (Ta stage) and lamina propria (T1 stage), accounting for approximately 75% of bladder cancers (2). MIBC invades the muscle (stage T2) or beyond (stages T3 and T4) (2). Up to 15% of patients with MIBC have a primary history of NMIBC, while the remaining patients are diagnosed with primary MIBC (4). Transurethral resection of the bladder tumor (TURBT) in combination with intravesical administration of chemotherapeutics or immunological pharmaceuticals is regarded as standard therapy for NMIBC (5). Treatments for MIBC consist of radical cystectomy, partial cystectomy, neoadjuvant therapy, cytotoxic chemotherapy, immune checkpoint inhibitors, or targeted therapies (2). However, a high proportion of patients with bladder cancer progress to high-grade or metastatic disease, with a 5-year progression rate ranging from 0.8% to 45% in various studies (2, 6). Therefore, effective therapeutics are needed to improve the prognosis of BLCA patients.

To date, cancer immunotherapy, especially immune checkpoint inhibitor (ICI) therapy, has gained considerable success as a cancer treatment (7–9). However, not all patients benefit from immunotherapy, both due to severe toxicity and high cost of treatment (9). In phase 3 IMvigor211 clinical trial of atezolizumab (PD-L1 inhibitor) targeting BLCA, the 24-mo survival rate was 23% with a median of 33 months of survival post-follow-up. Another phase 2 study, KEYNOTE-057, of pembrolizumab (PD-1 inhibitor) by Balar et al. showed that 39 of 96 patients (41%) with BCG-unresponsive carcinoma *in situ* in the bladder had a complete response at 3 months. 13 (13%) patients exhibited grade 3 or 4 treatment-related adverse events (10).

Tumor vaccines are another highly attractive alternative in cancer immunotherapy (11, 12). Tumor vaccines induce a sustained immune memory response that reactivates the patient’s immune system to destroy cancer cells at the initial moment of disease recurrence (13). Currently, tumor vaccines that are applied either for bladder cancer indication or in the clinical trial phase include BCG vaccine, MTBVAC (a live attenuated vaccine derived from mycobacterium tuberculosis), VPM1002BC (a modified BCG vaccine), PANVAC (a poxvirus vector-based vaccine derived from two viral vectors), among others (14–16). mRNA vaccines have recently attracted attention with their application against SARS-CoV-2 (17). Several mRNA-based cancer vaccines have been developed and registered for various clinical trial stages, including prostate cancer, glioblastoma, melanoma, and renal cell carcinoma (18). However, the application of mRNA vaccines for bladder tumors still remains to be explored.

The objectives of this study were to explore novel BLCA antigens for the development of mRNA vaccines and to map the immune landscape of BLCA in order to identify suitable patients for vaccination. Two genes associated with poor survival and antigen-presenting cell infiltration were identified and validated in several databases. Based on prognostically immune-related genes, we defined three immune subtypes between all cohorts and explored the characteristics of the three different subtypes. Our results can inform mRNA vaccine development and patient selection for BLCA vaccination.

## Methods

### Data source and processing

Gene expression data including mRNA count, Fragments Per Kilobase per Million (FPKM), somatic gene mutations, and DNA methylation data (450k methylation array data) of patients with bladder cancer, were retrieved from the Cancer Genome Atlas (TCGA; https://tcga-data.nci.nih.gov/tcga/). Gene copy number variation (CNV) DNA mutation data were collected from cBioPortal for Cancer Genomics (cBioPortal; http://www.cbioportal.org). Gene expression or mRNA array expression data of other BLCA cohorts were downloaded from Gene Expression Omnibus (GEO, https://www.ncbi.nlm.nih.gov/geo/) and ArrayExpress (https://www.ebi.ac.uk/arrayexpress/). To avoid the impact of batch effects, we only collected the BLCA cohorts (GSE13507, GSE32894, and E-MTAB-4321) with more than 100 samples with survival information for further analysis.

Next, we used the “Combat” algorithm in the “sva” package to reduce batch effects between all BLCA cohorts and produce a Meta cohort, which included TCGA-BLCA, GSE13507, GSE32894, and E-MTAB-4321 cohort.

Finally, mRNA sequencing data and clinical information about BLCA PD-L1 treatment were extracted from the datasets using the R package “IMvigor210CoreBiologies” (IMvigor 210 cohort), and the mRNA sequencing data and clinical information about melanoma PD-1 treatment cohort were collected from GEO (GSE78220).

### Gene differential expression analysis

To accurately select over-expressed genes and avoid data correction biases, we performed a differential analysis based on mRNA count data from the TCGA BLCA cohort and using the “DESeq2” algorithm. 1943 genes with log fold change (Log FC) > 2 and adjusted P < 0.05 were selected as over-expressed genes for further analysis.

### Survival analysis

Samples with no more than 30 days of overall survival (OS), and disease-free survival (DFS) were excluded from the TCGA cohort. The univariable Cox was used to define the hazard ratio (HR) for genes based on mRNA expression data (log_2_[TPM+1])) of the TCGA BLCA cohort. The statistical significance of survival data was tested by the log-rank test. A P < 0.05 was considered statistically significant.

### Identification of subtypes by immune-related genes in BLCA

A total of 1,811 immune-related genes extracted from the import database (https://www.immport.org/) were used for Kaplan-Meier and univariable Cox analysis of the TCGA BLCA cohort. Then, 128 prognostic immune-related genes were screened for non-negative matrix factorization (NMF) analysis. Silhouette coefficient and three-dimensional principal component analysis (3D PCA) were then used to validate the subtype assignments based on the mRNA expression data.

### Estimation of immune and stromal infiltration

To avoid bias of a single algorithm, the abundance of immune cell and stromal cell of BLCA sample were estimated by algorithms “xCell”, “CIBERSORT”, “Tumor Immune Estimation Resource (TIMER)”, “MCP-counter”, “EPIC”, and “quanTIseq” based on the R package “immunedeconv”. Single-sample Gene Set Enrichment Analysis (ssGSEA) was applied to assess the relative abundance of 23 immune cell types, stem cells, and active levels of other 15 signaling pathway ways for each sample. The list of genes corresponding to each immune cell type and the associated signaling pathways were obtained from recent publications (19, 20).

### Functional enrichment analysis

Biological characteristics of each immune subtypes were identified by GSEA analysis and Metascape (https://metascape.org/) based on differential expression of the identified genes between three subtypes.

### Building and publishing of R package “immcluster”

To predict the immune subtype of other BLCA cohorts not including in the meta cohort, we built a classifier based on XGBoost. First, 264 differential immune-related genes were identified by differential analysis (P < 0.01) based on training cohort (TCGA). Then, using the 10-fold cross-validation analysis of eXtreme Gradient Boosting (XGBoost), we optimized the parameters of the XGBoost model (max depth = 6, ta=0.4, and nround = 100) using the training cohort (TCGA). Next, we reduced batch effects between training and test cohorts using the “Combat” algorithm and predicted the immune subtype of each test cohort using the previously trained XGBoost model and TCGA cohort after batch correction. Finally, to facilitate other researchers using the model and validating the proposed immune subtypes, we built the R package “immcluster” and released it on Github (https://github.com/ZylRpackage2022/immcluste).

### Drug sensitivity analysis

The response of immunotherapy of the Meta cohort was predicted by Tumor Immune Dysfunction and Exclusion (TIDE, http://tide.dfci.harvard.edu/). The IC50 of chemotherapy of the TCGA BLCA cohort was predicted by R package “pRRophetic”.

### Immunohistochemistry

BLCA tissue with adjacent normal bladder tissue microarray (HBlaU050CS01) was purchased from Shanghai Outdo Biotech Co., Ltd., which contained 50 cases. Immunohistochemical staining of microarrays was performed by Wuhan Servicebio Technology Co., Ltd. (Wuhan, China). The primary antibodies used were IGF2BP2 (Proteintech, Chicago, USA; catalog no. 11601-1-AP; used at a 1:1000 dilution), and MMP9 (Servicebio, Wuhan, China catalog no. GB11132-2, using at a 1:200 dilution).

Histology score (H-score) analysis was used to assess the staining intensity using the AIpathwell Software (Servicebio Technology Co., Ltd.). The following formula was applied: 


$$\begin{array}{c} \text{H}-\text{score}=\sum (Pi\times \text{i})=(\text{percentage of weak intensity cells}\times 1)\\ \;\;\;+(\text{percentage of moderate intensity cells}\times 2)+\\ \;\;\;\;(\text{percentage of strong intensity cells}\times 3)\\ \;\;(\text{where Pi represents the percentage of positivity},\text{ and i represents intensity score}) \end{array}$$

### Cell cultures and reagents

The mediums used for cell culture were listed in the Table S1. SiRNA for IGF2BP2 and negative control (NC) were provided by Genepharma (Shanghai, China). The siRNA sequences were shown in **Table S1**.

### RNA extraction and RT-qPCR methods

The kit for RNA extraction and RT-qPCR was listed in **Table S1**.

Relative expression levels of IGF2BP2 were calculated using the comparative *Ct* (ΔΔ*Ct*) method, where *Ct* was the cycle threshold number and normalized to GAPDH. The primer sequences for RT-qPCR were shown in the **Table S1**.

### Scratch assay method

After 48h of transfection, UMUC3 and T24 cells were seeded into 6-well plates (1×10^6^ cells/well). When cells reached over 80-90% confluence, a single wound was made with a sterile plastic 20 μl pipette tip. After removing cellular debris, fresh serum-free medium was added. Next, the wound was photographed at 0, 6, and 24 hours after scraping. The cell migration rate was calculated by the formula as follows:

Migration rate (%) = (original distance − measured distance)/original distance×100%.

### Western blot

The following antibodies were used: rabbit polyclonal anti-IGF2BP2 (Proteintech, Chicago, USA; catalog no. 11601-1-AP), and mouse monoclonal anti-β-actin (Proteintech, Chicago, USA; catalog no. 66009-1-Ig).

### Transwell assay

The 24-well transwell chamber was used (Corning Inc.) to determine the cell migration capacity. After 48 h of transfection, UMUC3 and T24 cells (1x10^5^ cells/well) were seeded into the upper chamber with a serum-free medium, and 500 μl medium with 10% FBS was added to the lower chamber to act as a chemoattractant. After incubation at 37°C for 48 h, migrated cells were fixed by 4% formaldehyde and then stained using 0.5% crystal violet.

### Statistical analysis

Mann-Whitney U test was used to compare the two groups with non-normally distributed variables. As a nonparametric method, the Kruskal-Wallis test was adopted to compare multiple groups. Contingent variables were analyzed with the chi-square test or Fisher’s exact test. Spearman’s test was conducted to analyze the correlation between gene expression and immune cell abundance. Students t-test was used to compare the differences of the staining intensity between the normal tissue and bladder cancer groups after normality testing. The analyses were performed using R software (Version 4.1.2, https://www.r-project.org/) and Statistical Package for the Social Sciences Software (SPSS, version 23.0, Chicago, IL, USA.) The difference was considered statistically significant with P < 0.05.

## Results

### Identification of potential antigens in BLCA

**Figure 1:**
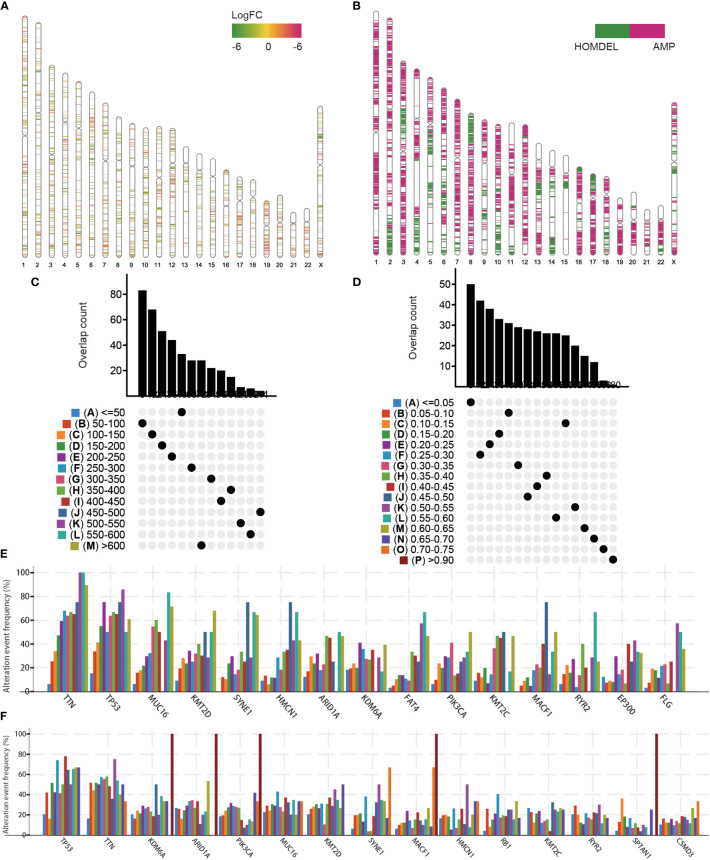
Identification of potential tumor antigens in BLCA. **(A)** Chromosomal distribution of the overexpressed genes in BLCA. **(B)** Chromosomal distribution of the aberrant copy number genes in BLCA. Overlapping mutated genes distributed in the fraction genome altered group **(C)** and mutation count group **(D)** were shown. Genes with the highest frequency in the fraction genome altered groups **(E)** and mutation count groups **(F)** were individually shown.

To detect potential tumor antigens of BLCA, we first identified 6,739 overexpressed genes between normal and tumor tissues and 11,978 genes exhibiting copy number amplification (amplified rate > 1%). Their distributions on the chromosomes were shown in **Figures 1A, B**. Then, a total of 9,413 mutated genes (mutation rate > 1%) were screened by altered genome fraction and mutation counts (**Figures 1C, D**). The most frequently mutated 15 genes were illustrated in **Figures 1E, F**. Mutational analysis identified TIN, TP53, MUC16, KMT2D, SYNE1, HMCN1, ARID1A, KDM6A, PIK3CA, KMT2C, MACF1, and RYR2 as the most frequently mutated genes in terms of both altered genome fraction and mutational counts. Overall, 196 overlapping genes from overexpressed, highly amplified, and frequently mutated genes included in the Meta cohort were identified in the TCGA dataset that could serve as potential tumor antigens (**Table S2**).

### Identification of tumor antigens associated with BLCA prognosis and antigen presenting cells

**Figure 2:**
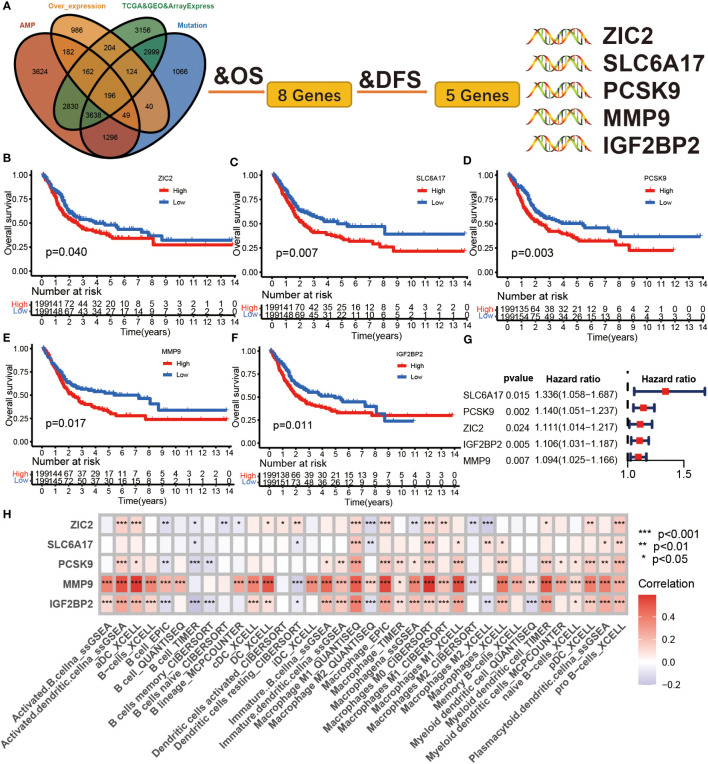
Identification of tumor antigens associated with clinical outcome in BLCA. **(A)** Tumor antigens significantly associated with OS and DFS from over expression, highly amplified, and mutated overlapping genes. **(B-F)** Kaplan-Meier survival curve analysis for ZIC2 **(B)**, SLC6A17 **(C)**, PCSK9 **(D)**, mmp9 **(E)**, and IGF2BP2 **(F)** genes in the TCGA BLCA cohort. The log-rank test was used to determine the statistical significance of the differences, and P< 0.05 was considered significant. **(G)** The univariable COX analysis of five potential tumor antigens in TCGA BLCA cohort. **(H)** The correlation between APCs and five tumor antigens in the Meta cohort.

The 196 aforementioned overlapping genes were used for survival analysis to develop prognostically relevant antigens. Eight genes were related to overall survival (OS) of BLCA patients, where five genes showed a strong correlation with DFS (Disease-free survival) (**Figure 2A**; **Table S3**). The survival curves in **Figures 2B-F** indicated that elevated expression of ZIC2, SLC6A17, PCSK9, MMP9, and IGF2BP2 in tumor tissues were associated with poor prognosis compared to the low expression group. The results of univariate COX regression analysis indicated that all five genes were risk factors for bladder cancer (HR > 1) (**Figure 2G**). In summary, these five genes were screened as prognostically-relevant antigens in BLCA.

**Figure 3:**
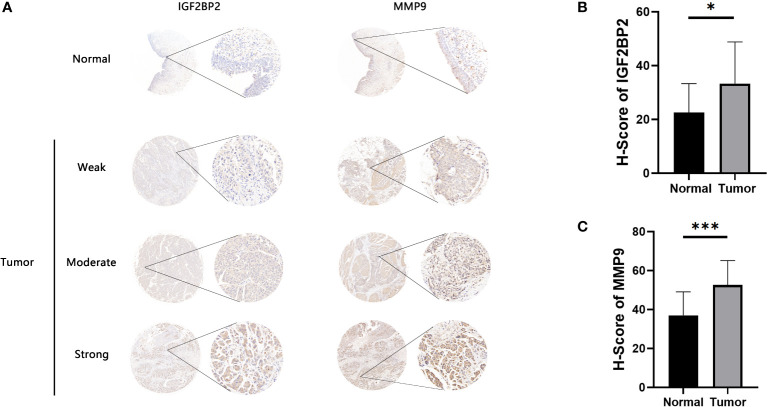
Immunohistochemical evaluation of IGF2BP2 and MMP9. **(A)** Weak, moderate, and strong immunohistochemical staining of IGF2BP2 shown respectively in normal and BLCA samples. **(B)**The H-scores of IGF2BP2 in BLCA tissues compared with normal tissues. **(C)** The H-scores of MMP9 in BLCA tissues compared with normal tissues. *P < 0.05, ***P < 0.001.

Antigen-presenting cells (APCs), including dendritic cells (DCs), B cells, and macrophages, process and deliver antigens to T lymphocytes, which initiate an adaptive immune response. We next evaluated the relationship between the five overexpressed genes and antigen-presenting cell (APC) abundance using seven bioinformatic methods. As shown in **Figure 2H**, MMP9 and IGF2BP2 were positively correlated with levels of several APCs. We also explored the correlations between five overexpressed genes and markers of APCs through Spearman rank correlation and found that MMP9 and IGF2BP2 were significantly related to expressions of markers (**Table S4**). The results of tissue microarray-based immunohistochemical staining were shown in **Figure 3A**. The expression of IGF2BP2 and MMP9 was upregulated in tumor tissues compared with adjacent normal tissues (P<0.05) (**Figures 3B, C**).

Since MMP9 has been reported extensively in the field of oncology including BLCA, we chosen IGF2BP2 for further functional studies (21–24). As illustrated in **Figure S1A**, the relative expression levels of IGF2BP2 in common bladder cell lines were examined by RT-qPCR. Compared to urothelial cell line (SV-HUC-1 cells), the expression levels of IGF2BP2 were found to be relatively high in the bladder cancer cell lines UMUC3 and T24 cells. To further investigate the function of IGF2BP2 in bladder cells, we knocked down IGF2BP2 by specific interfering RNA (siRNA) in UMUC3 and T24 cells. Then, RT-qPCR and western blot analyses were performed to validate the knockdown efficiency of siRNA (**Figures S1B, C**). Compared to the NC group, the migration ability of the BLCA cells in the siRNA2 group was reduced significantly after interference with IGF2BP2 expression (**Figures S1D, E**). Taken together, two tumor antigens (MMP9 and IGF2BP2) were identified as promising candidates for developing mRNA vaccines against BLCA.

### Definitions of the three immune subtypes of BLCA

**Figure 4:**
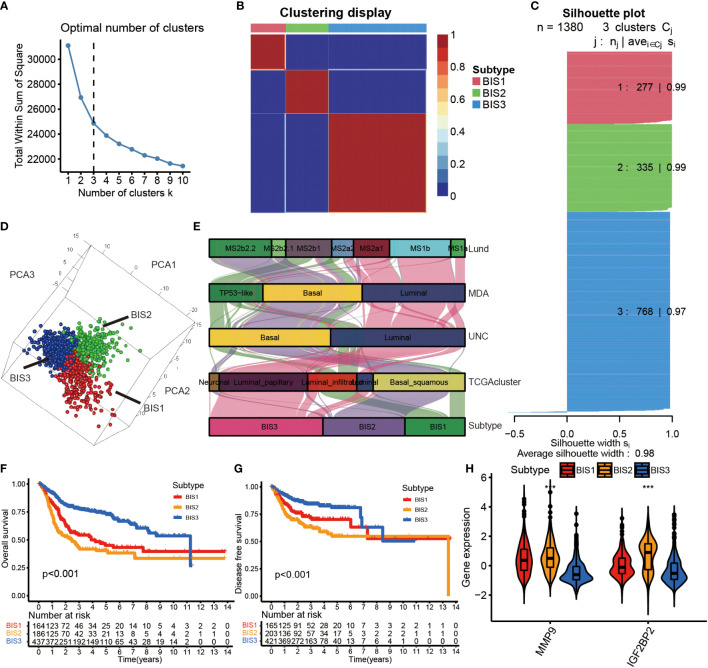
Identification of immune subtypes in BLCA. **(A)** The Total Whin Sum of Square of NMF analysis with varying cluster number (k=1-10). **(B)** Heatmap representing the consensus matrix in the Meta cohorts. **(C)** The Silhouette index of each sample after BLCA samples were divided into three clusters by NMF analysis. **(D)** Stratification into three subtypes validated by 3D PCA analysis in the Meta cohorts. **(E)** Correlation between immune subtypes and other molecular subtypes confirmed by previous studies in the TCGA cohort. **(F)** Survival analysis of OS for three subtypes in the Meta cohorts. **(G)** Survival analysis of DFS for three subtypes in the Meta cohorts. The log-rank test was used to determine the statistical significance of the differences, and P< 0.05 was considered significant. **(H)** IGF2BP2 and MMP9 gene expression levels in three subtypes. ***P < 0.001.

Initially, a total of 1,811 immune-related genes were extracted from the import database to screen prognostically-related genes in the TCGA database. Consensus clustering was performed using Nonnegative Matrix Factorization (NMF) analysis based on the 128 prognostically immune-related genes in Meta cohort. Using the Total Within Sum of Square index (**Figure 4A**), we identified the optimal cluster number (n = 3) of BLCA patients in the Meta cohort and defined three immune subtypes, referred to as BIS1-BIS3 (**Figure 4B**). Silhouette plot (average silhouette width = 0.98) and 3D PCA analysis indicated distinct separations between the three subtypes (**Figures 4C, D**). We next compared the classifications defined in this study with previous classifications from the TCGA cohort (25). We found that bladder cancers of subtype BIS1 were mainly enriched in TP53-like and MS2b1 subtypes, samples of subtype BIS2 were mainly enriched in Basal and MS2b2, those with subtype BIS3 were primarily enriched in Luminal, MS1b and MS2a1 subtypes (**Figure 4E**). Subsequently, we explored the relationship between survival and BLCA subtype. BIS3 (n = 437) was associated with better prognosis (log-rank test, P< 0.001, **Figures 4F**, **G**), while BIS1 (n = 164) and BIS2 (n = 186) had poorer survival probability in the Meta cohort. Additionally, consistent results were obtained in each cohort (TCGA OS: log-rank test, P< 0.001; TCGA DFS: log-rank test, P = 0.002; GSE13507: log-rank test, P = 0.009; GSE32894: log-rank test, P< 0.001; E-MTAB-4321: log-rank test, P< 0.001) (**Figure S2**), indicating the reproducibility and stability of the subtypes identified in this study. Finally, we explored MMP9 and IGF2BP2 expression levels in the three subtypes and found that the two potential tumor antigens were lowest in BIS3 and highest in BIS2 (Kruskal-Wallis test, P< 0.001) (**Figure 4H**). In conclusion, immunotyping can be used to predict patient prognosis, and patients of the BIS3 subtype could potentially have better prognoses.

### The clinical, cellular and immune infiltration characteristics of BLCA tumor with the three subtypes

Subsequently, the clinical features of the three immune subtypes were investigated in the TCGA cohort (**Figure S3**). BIS3 was less malignant, considering pathological stage and histologic grade (x^2^ test, P< 0.001) compared to the other two subtypes, consistent with survival outcome. In contrast, age (x^2^ test, P = 0.2433) and gender (x^2^ test, P = 0.5091) showed no significant difference among three subtypes (**Table S5**).

**Figures S4A, B** depicted the activated and inhibited pathways in the three subtypes based on the Kyoto Encyclopedia of Genes and Genomes (KEGG) functional enrichment analysis. Additionally, we explored the signaling pathways critical for tumorigenesis and progression in three subgroups and found that the three groups were inconsistently expressed in all pathways (**Figure S4C**). To address the self-regenerating properties, we compared the ssGSEA scores of stem cell gene sets in three subtypes, and the results showed that BIS2 had the highest score among the three groups, followed by BIS1 and BIS3 (**Figure S4D**).

**Figure 5:**
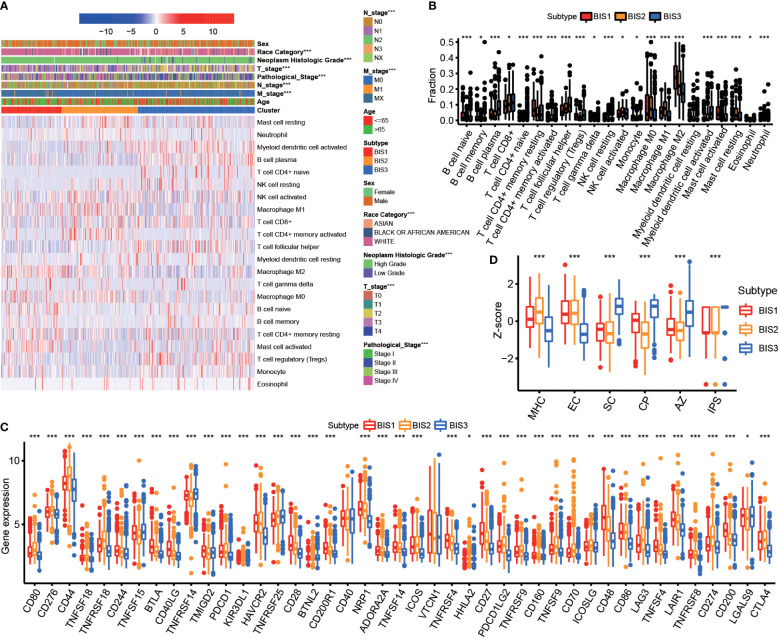
Immune characteristics of three subtypes. **(A)** Heatmap of immune cell fraction among three subtypes in TCGA BLCA cohort. **(B)** Boxplot of immune cell fraction among three subtypes in TCGA BLCA cohort. **(C)** Immune checkpoint of three subtypes in Meta cohort. **(D)** Immunophenogram for the visualization of the parameters determining immunogenicity. Boxplot showed the immune score of three subtypes in TCGA BLCA cohort (MHC, major histocompatibility complex-related molecules; EC, effector cells; SC, suppressor cells; CP, checkpoints or immunomodulators; AZ, averaged z-score; IPS, immunophenoscore). *P < 0.05, **P < 0.01, ***P < 0.001.

Since response to mRNA vaccines correlates with tumor immune status in the tumor microenvironment, the CIBERSORT algorithm was used to evaluate the proportions of 22 types of immune cell subpopulations among three subtypes (**Figures 5A, B**) (26). BIS3 displayed higher abundance of CD8+ T cells, B cells, and monocytes, while BIS3 showed lower proportions of immune inhibitory cells such as M2 macrophage. Therefore, we concluded that the BIS3 subtype exhibited an “active” immune phenotype, while BIS1 and BIS2 subtypes had a “suppressive” immune phenotype.

**Figure 5C** showed the expression of certain co-inhibitory and co-stimulatory molecules in the three subtypes. We found that the majority were differentially expressed in three subgroups.

Next, we characterized the immunogenicity of tumor subsets by immunophenoscore (IPS) (27) and visualized using an immunophenogram (**Figure 5D**). The results showed that BIS3 had a higher score in checkpoints or immunomodulators (CP) and IPS, while BIS1 and BIS2 had a higher score in major histocompatibility complex-related molecules (MHC molecules). These results indicated that the immune subtypes mirrored BLCA immune status and could stratify patients by suitability for mRNA vaccination. We hypothesized that mRNA vaccines targeting MMP9 and IGF2BP2 could induce immune infiltration in the tumor microenvironment of patients with immunologically “suppressive” BIS1 and BIS2 tumors.

### The molecular and multi-omics characteristics of BLCA tumors within the three subtypes

**Figure 6:**
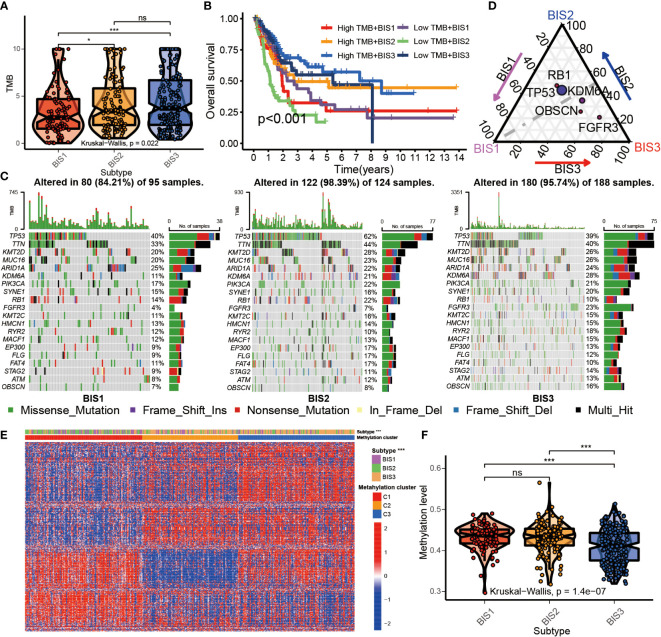
Multi-omics characteristics among three subtypes. **(A)** Boxplot of TMB in three subtypes in TCGA BLCA cohort. **(B)** Survival analysis for three subtypes and high/low TMB groups in the TCGA BLCA cohorts. **(C)** The waterfall plot of top 20 mutated genes among three subtypes in the TCGA BLCA cohorts. **(D)** Triangle plot of differential gene mutation among three subtypes in the TCGA BLCA cohorts. (Fisher’s test, P< 0.05) **(E)** Heatmap of top 500 variance genes methylation level in three subtypes. **(F)** The average methylation level of immune-related genes in three subtypes in TCGA BLCA cohorts. *P < 0.05, ***P < 0.001; ns, no significance.

High tumor mutational burden (TMB) is correlated with neo-antigen expression, which is presented by MHC proteins to T-cells and induce an effector T-cell response (28). High TMB also correlates with improved response to immune checkpoint inhibitors (ICIs) and mRNA vaccination (29). BLCA belonging to subtypes BIS3 and BIS2 had a significantly higher TMB compared to patients with BIS1 in TCGA cohort (**Figure 6A**; P< 0.05). When TMB was included as a variable, differences in overall survival between the three subtypes was highly significant (P< 0.001). BIS3 with high TMB showed the best prognosis, and BIS2 with low TMB displayed the worst prognosis (**Figure 6B**), consistent with our previous findings (**Figures 4F, G**). Among the three immune subtypes, BIS2 had the highest mutation rate (98.39%), followed by BIS3 (95.74%), and BIS1 (84.21%). The landscape of the 20 most frequently mutated genes between the three subtypes was displayed in **Figure 6C**. TP53 was the most frequently mutated gene in all three subgroups (**Figures 6C, D**; **Table S6**, P< 0.05). Overall, we found that TMB had a clear relationship with our three previously defined immune subtypes of BLCA, which remained to be explored further.

Related studies have found that DNA methylation can affect the immune status of BLCA (30, 31). To explore the relationship between DNA methylation in whole-genome and immune subtypes, we performed NMF analysis based on the top 500 genes of variance of DNA methylation level and characterized the DNA methylation status of the three immune subtypes (**Figure S5**). The results showed that BIS3 was corresponded with the cluster2 and cluster3 of DNA methylation subtypes (**Figure 6E**). BIS3 had reduced whole-genome DNA methylation compared to BIS1 and BIS2 (**Figure 6F**). This result suggested that the DNA methylation could directly affect the immune status of BLCA.

### Developing a classifier to predict immune subtypes in the BLCA cohorts

**Figure 7:**
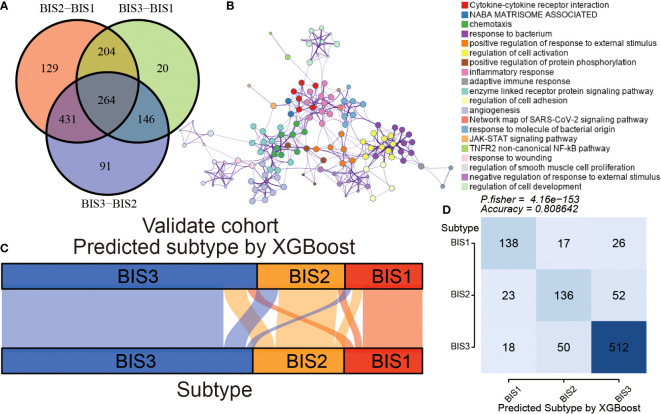
Construction and validation of an immune subtype classifier. **(A)** Venn diagram showing differential expression of immune-related gene between three subtypes in TCGA BLCA cohort. **(B)** The Metascape analysis of differential expression of immune-related genes. The alluvial diagram **(C)** and Fisher’s test **(D)** of three subtypes samples in the validation cohort.

To make our immune subtypes reproducible and generalizable, we developed a classifier to predict immune subtypes in new samples. First, we performed pairwise differential analysis across the immune subtypes in the training cohort (TCGA) based on 1,811 immune-related genes. A total of 264 overlapping differential expressed genes were prioritized as immune subtype-related genes for further analysis (**Figure 7A**; **Table S7**). Metascape analysis revealed that these genes were closely associated with the cell immune reactivity (**Figure 7B**; **Table S8**). Next, the XGBoost algorithm was used to create a classifier to predict immune subtypes, performing parameter optimization 10-fold cross-validation (**Figure S6**). Subsequently, we validated the accuracy of our classifier using the”immcluster” R package, applied to the TCGA (training) cohort, Meta cohort (excluding TCGA; testing cohort), (**Figures 7C, D**), and three independent cohorts (**Figure S7**; **Table S9**). Our classifier had accuracies of 1.000, 0.809, 0.803, 0.825, and 0.841, respectively, showing the robust performance of our package. Importantly, the “immcluster” R package can eliminate batch effects of data from multiple datasets to improve accuracy and predictive power on validation datasets.

### Immune subtypes and classifier to help antitumor therapy in BLCA

**Figure 8:**
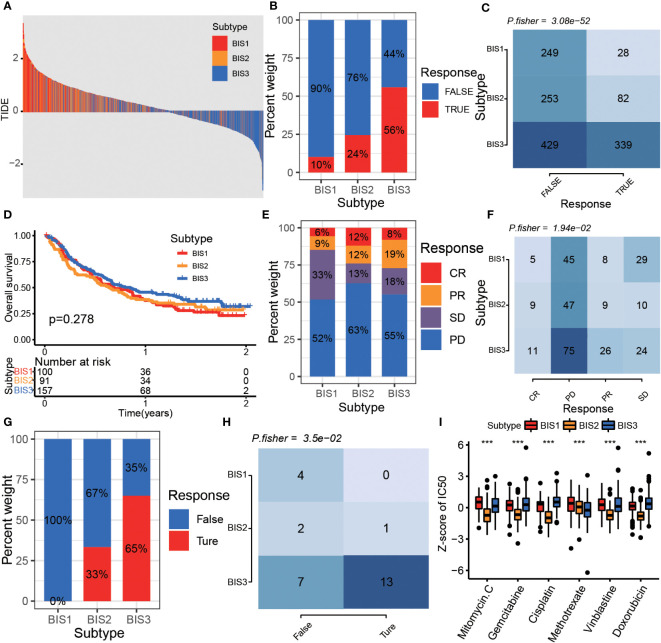
The drug sensitivity of different subtypes. **(A)** The distribution of TIDE score among the three subtypes in Meta cohort. The proportion **(B)** and Fisher’s test **(C)** of immunotherapy response samples in different subtypes in Meta cohort. **(D)** Survival analysis for three subtypes in the IMvigor210 cohort. The proportion **(E)** and Fisher’s test **(F)** of immunotherapy response samples in different subtypes in the IMvigor 210 cohort. The proportion **(G)** and Fisher’s test **(H)** of immunotherapy response samples in different subtypes in GSE78220 cohort. **(I)** The boxplot of z-score of chemotherapy drug IC50 between three subtypes in the TCGA BLCA cohort. ***P < 0.001.

Since immune characteristics of tumors correlate with treatment efficacy, we further explored the responsiveness of different immune subtypes to antitumor therapy. First, we used the TIDE algorithm to select patients who showed better responses to immune checkpoint blockade (ICB) (32). The results showed that patients in the BIS3 subtype had a lower TIDE score than those in BIS1 and BIS2, and BIS3 had the highest proportion of patients who responded to ICB, followed by BIS2 and BIS1 (**Figures 8A-C**, P< 0.001). We further validated the TIDE predictions in a large phase 2 trial (IMvigor210) (33), which investigated the clinical activity of PD-L1 blockade in metastatic urothelial cancer (**Figures 8D-F**). We found that BIS3 had the highest proportion of individuals responding to PD-L1 (27%), followed by BIS2 (24%), and BIS1 (15%), consistent with the TIDE predictions. In addition, we explored the response of patients within the three immune subtypes to PD-1 blockers in a GSE78220 (melanoma) cohort (34) and found a similar trend to the IMvigor210 cohort (**Figures 8G, H**; P< 0.05).

Finally, we predicted the IC50 of multiple chemotherapeutics in the TCGA BLCA cohort. The results indicated that the BIS2 subtype was most sensitive to drugs including mitomycin C, gemcitabine, cisplatin, vinblastine, and doxorubicin, and the BIS3 subtype was most sensitive to methotrexate treatment (**Figure 8I**, P< 0.001).

## Discussion

Successful identification of tumor-associated antigens is the basis for vaccine development. Proteins dysregulated by genetic and epigenetic aberrations in tumor cells, when recognized by the immune system to attack cancer cells, can be classified as tumor antigens (35). In the present study, we identified five genes from overexpressed, highly amplified, and frequently mutated genes, which were significantly associated with worse prognosis in patients with BLCA in the Meta cohort and other cohorts. Further, we found that gene expression of MMP9 and IGF2BP2 was positively correlated with levels of several APCs, which processed tumor antigens and presented them to CD8+T cells. Additionally, we validated the expression of these two genes in clinical samples and found that MMP9 and IGF2BP2 genes were highly expressed in bladder cancer tissues compared to adjacent normal tissues, which meant they had the potential for the development of tumor vaccines. Though further preclinical evaluation and validation are still required, several previous studies have considered the potential of these two antigens as targets for mRNA vaccine targeting of BLCA. Owyong et al. reported that MMP9 played a key role in the early metastatic niche of tumorigenesis and promoted lung colonization of circulating tumor cells. In the MMTV-PyMT model, blocking the active form of MMP9 with a monoclonal antibody inhibited endogenous and experimental lung metastases (36). Andecaliximab (GS-5745, a monoclonal antibody targeting MMP9) had been evaluated in several clinical trials for indications including advanced gastric and gastroesophageal junction adenocarcinoma (37) and advanced pancreatic adenocarcinoma (38). IGF2BP2, an N6-methyladenosine (m6A) reader, participates in multiple biological processes by interacting with different RNAs (39). Overexpression of IGF2BP2 has been found to confer shorter survival and poor prognosis in multiple cancers (39), including breast cancer (40), hepatocellular carcinoma (41), and pancreatic ductal adenocarcinoma (42, 43).

Since the therapeutic response to an mRNA vaccine may be limited to a small subset of patients, it is essential to screen suitable populations for their suitability to vaccination (44). Using prognostically immune-related genes, we classified bladder cancer into three immune subtypes (BIS1, 2, and 3). Patients in BIS3 had the best survival among the three subtypes, while patients in BIS2 had shortened survival compared with those in other subtypes, suggesting that the immunophenotype can serve as a prognostic factor for BLCA. MMP9 and IGF2BP2 expression were lowest in BIS3 and highest in BIS2. In addition, immunophenoscore results of CP and IPS were highest in BIS3, while BIS1 and BIS2 had a higher score in MHC molecules.

Patients with subtype BIS3 and BIS2 had a substantially higher TMB compared to patients with BIS1 in TCGA cohort. These results indicated that patients in the BIS2 subgroup were more likely to be responsive to mRNA vaccines targeting MMP9 and IGF2BP2, while immune checkpoint inhibitors were more suitable for patients in BIS3. Recently, McCann K et al. demonstrated that vaccination in non-small cell lung cancer with low mutational load was feasible and could be effective (45). Therefore, clinical trials of vaccination against the BIS2 subgroup, which had high MMP9 and IGF2BP2 expression and low TMB, is a promising future direction.

Compared to other types of vaccines, mRNA vaccines have several advantages. The risk of insertional mutations by integration into the host cell genome is unlikely to be a concern for mRNA vaccines. In addition, the manufacturing process of mRNA does not require cell culture or toxic chemicals; thus, mRNA vaccines are considered to be relatively safe (46). mRNA vaccines have the advantage of encoding different proteins or long peptides, enabling a wide range of polyclonal immune responses, thus avoiding possible immune escape owing to antigen loss or the restriction to a certain HLA molecule (47). Meanwhile, mRNA vaccines could deliver multiple antigens simultaneously, improving the efficiency and effectiveness of treatment (48, 49). Recently, several technical obstacles have been solved, including stability, delivery, and immunogenicity of mRNA, and efficient delivery has been achieved *in vivo* (46). In contrast to traditional protein subunit and viral vaccines, mRNA vaccine production is rapid and relatively simple in production and manufacturing (46). In this study, we focused on MMP9 and IGF2BP2 genes, which were both elevated in BIS1 and BIS2 subtypes. Therefore, these two genes could be made into a bivalent vaccine composition, which could expand the vaccine population and enhance immune responses (48, 49). Alternatively, tumor-targeting mRNA vaccines could be combined with traditional chemotherapeutic agents or immune checkpoint inhibitors. A phase II trial investigated the combination of TriMixDC-MEL (autologous monocyte-derived dendritic cells electroporated with synthetic mRNA) and ipilimumab (CTLA-4 blocking monoclonal antibody) in patients with pretreated advanced melanoma. The results showed that this combination achieved a high rate of durable tumor responses in patients who achieved complete responses (50).

Several reports have explored the application of mRNA vaccines to urinary tumors, including bladder tumor (51). Gui C et al. established a ferroptosis-induced tumor microenvironment landscape in bladder cancer and identified six genes as potent antigens for developing an anti-BLCA mRNA vaccine (52). Wang G et al. identified AP2S1, P3H4, and RAC3 as three candidate genes of tumor-specific antigens in bladder cancer using the TCGA BLCA cohort and GSE13507 datasets (53). In contrast to these studies, we integrated four cohorts into a Meta cohort at the outset, including TCGA BLCA, GSE13507, GSE32894, and E-MTAB-4321 cohorts, which enhanced the robustness of our study. In addition, we validated the expression of MMP9 and IGF2BP2 in clinical samples, which was not done in other studies. Importantly, in order to perform immunotyping in new samples, we developed a classifier and released it for public use hosted on Github, which could eliminate batch effects from new datasets and make our immune subtypes reproducible and generalizable for other researchers. Furthermore, we validated the effect of various treatments on different subgroups in new datasets stratified by our classifier (IMvigor210 and GSE78220), which extended the value of the study.

## Conclusions

In conclusion, this study identified IGF2BP2 and MMP9 as potential antigens for mRNA vaccine development targeting BLCA, which could highly benefit patients specifically in the BIS2 subtype. The findings in this study provide a theoretical foundation for developing mRNA vaccines against BLCA, predicting patient prognosis, and defining suitable patient populations for vaccination. mRNA vaccination therapy should be further explored in prospective clinical trials.

## Data availability statement

The datasets presented in this study can be found in online repositories. The names of the repository/repositories and accession number(s) can be found in the article/**Supplementary Material**.

## Ethics statement

The studies involving human participants were reviewed and approved by the ethics committee of Shanghai OUTDO Biotech Corporation Limited. The patients/participants provided their written informed consent to participate in this study.

## Author contributions

XH, MZ and XW conducted this research study and revised the manuscript. XZ, YZ and LZ designed the research processes, exported the figures, wrote the first draft of the manuscript, and performed the verification experiments. JW and JL analyzed the experimental data. All authors contributed to the article and approved the submitted version.

## Acknowledgments

The authors would like to express their gratitude to EditSprings (https://www.editsprings.cn) for the expert linguistic services provided.

## Conflict of interest

The authors declare that the research was conducted in the absence of any commercial or financial relationships that could be construed as a potential conflict of interest.

## Publisher’s note

All claims expressed in this article are solely those of the authors and do not necessarily represent those of their affiliated organizations, or those of the publisher, the editors and the reviewers. Any product that may be evaluated in this article, or claim that may be made by its manufacturer, is not guaranteed or endorsed by the publisher.

## Supplementary material

Supplementary Figure 1 Experimental verification of IGF2BP2. **(A)** Relative expression of IGF2BP2 in bladder cell lines. **(B)** The knockdown efficiency of siRNA in UMUC3 and T24. **(C)** The knockdown efficiency of siRNA2 in UMUC3 and T24. Transwell migration assay **(D)** and wound healing assay **(E)** show that interference with IGF2BP2 expression inhibits the migration of UMUC3 and T24.

Supplementary Figure 2 Survival analysis between three subtypes in GSE13507 **(A)**, GSE32894 **(B)**, TCGA OS **(C)**, TCGA DFS **(D)**, and E-MTAB-4321 **(E)** cohorts.

Supplementary Figure 3 The proportion of different age **(A)**, sex **(B)**, race category **(C)**, T stage **(D)**, N stage **(E)**, M stage **(F)**, neoplasm histologic grade **(G)**, and pathological stage **(H)** in three subtypes in TCGA cohort.

Supplementary Figure 4 The molecular characteristics of three subtypes. The keeg function enrichment analysis identifies the activated **(A)** and inhibited pathways **(B)** in each immune subtype. **(C)** The boxplot of ssGSEA score of signaling pathways between three immune clusters. **(D)** The boxplot of ssGSEA score of stem cell gene set between three immune clusters.

Supplementary Figure 5 Identification of methylation subtypes in BLCA. **(A)** The Total Whin Sum of Square of NMF analysis with different clusters number (k=1-10). **(B)** Stratification into three subtypes validated by PCA analysis in the Meta cohorts. **(C)** Heatmap representing the consensus matrix in the Meta cohorts. **(D)** The Silhouette index of each sample while BLCA samples were divided into three clusters by NMF analysis.

Supplementary Figure 6 Example R script of using ”immcluster.”

Supplementary Figure 7 The alluvial diagram of three subtypes predicted by XGBoost in TCGA **(A)**, E-MTAB-4321 **(B)**, GSE13507 **(C)**, and GSE32894 **(D)** cohorts. Survival analysis between three subtypes predicted by XGBoost in E-MTAB-4321 **(E)**, GSE13507 **(F)**, and GSE32894 **(G)** cohorts.

Supplementary Table 1 The mediums used for cell culture, the kit for RNA extraction and RT-qPCR, siRNA sequences, and RT-qPCR primer sequences.

Supplementary Table 2 The over-expression genes, mutation genes, and AMP genes.

Supplementary Table 3 Survival analysis of OS/DFS for overlapping genes.

Supplementary Table 4 Correlation between potential tumor antigens and APCs or APC markers.

Supplementary Table 5 Fisher’s test of different age, sex, race category, T stage, N stage, M stage, neoplasm histologic grade, and pathological stage in three subtypes in the TCGA cohort.

Supplementary Table 6 Differential gene mutation in the three subtypes.

Supplementary Table 7 Differential expression of immune-related genes in three subtypes.

Supplementary Table 8 Metascape analysis of differential immune-related genes.

Supplementary Table 9 Model effectiveness of XGBoost in TCGA, E-MTAB-4321, GSE13507, and GSE32894 cohorts.
