# Supplementary figures and images for: Exploitation of tumor antigens and construction of immune subtype classifier for mRNA vaccine development in bladder cancer
Source: Front Immunol. 2022 Nov 16;13:1014638. doi: 10.3389/fimmu.2022.1014638 (PMC9769457; doi:10.3389/fimmu.2022.1014638)

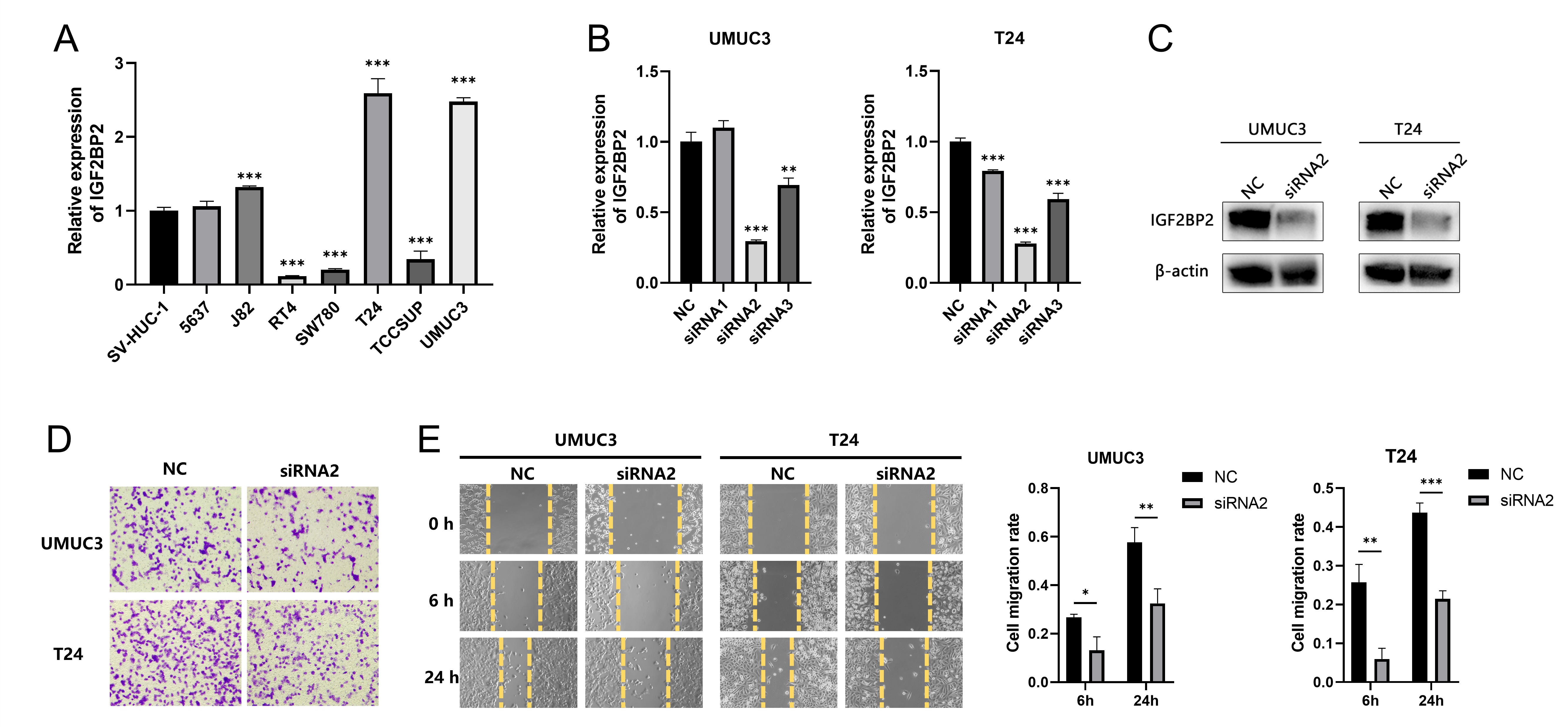

Supplement: Supplementary Figure 1 — Experimental verification of IGF2BP2. (A) Relative expression of IGF2BP2 in bladder cell lines. (B) The knockdown efficiency of siRNA in UMUC3 and T24. (C) The knockdown efficiency of siRNA2 in UMUC3 and T24. Transwell migration assay (D) and wound healing assay (E) show that interference with IGF2BP2 expression inhibits the migration of UMUC3 and T24. [file Image_1.tif]

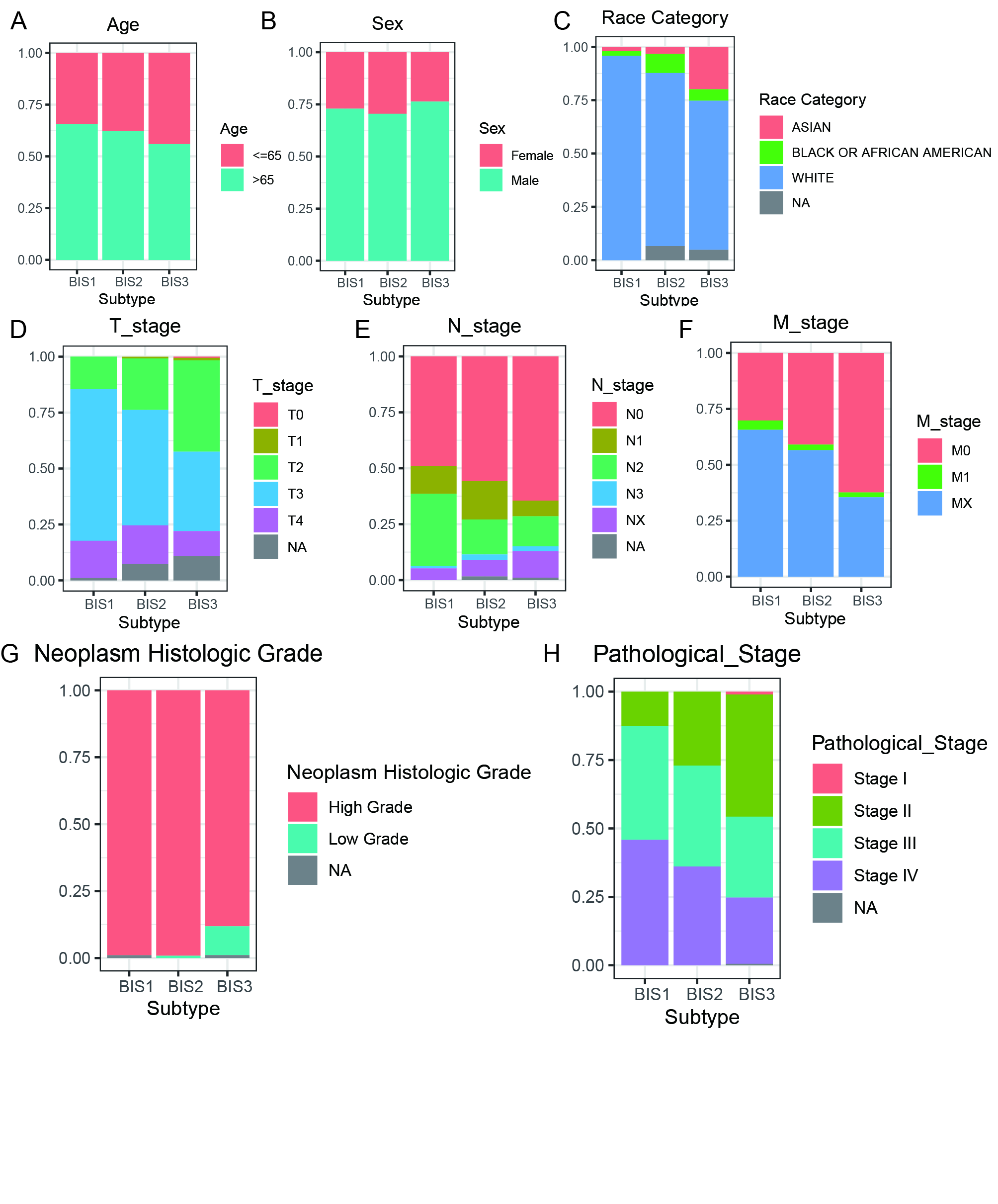

Supplement: Supplementary Figure 2 — Survival analysis between three subtypes in GSE13507 (A), GSE32894 (B), TCGA OS (C), TCGA DFS (D), and E-MTAB-4321 (E) cohorts. [file Image_3.tif]

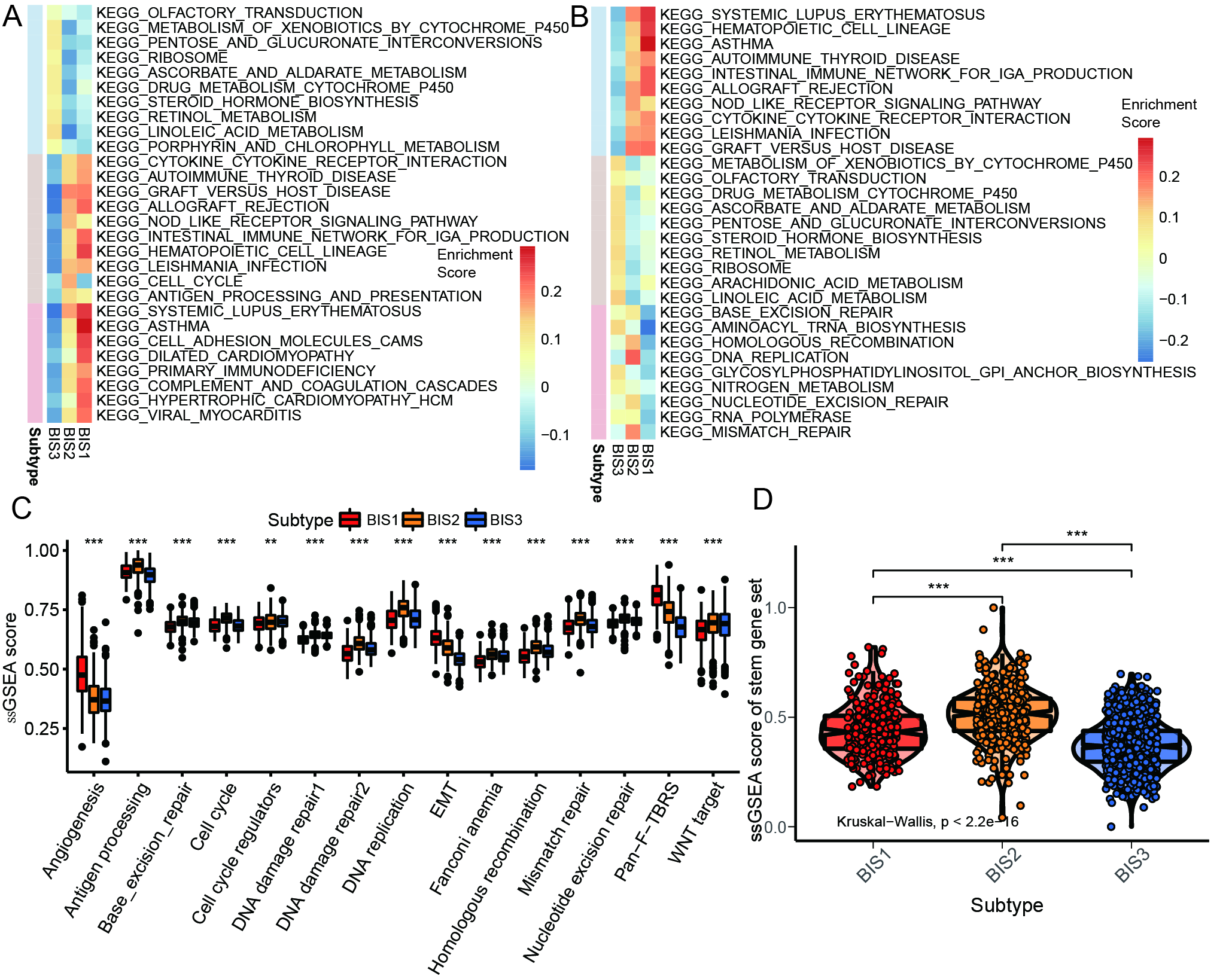

Supplement: Supplementary Figure 3 — The proportion of different age (A), sex (B), race category (C), T stage (D), N stage (E), M stage (F), neoplasm histologic grade (G), and pathological stage (H) in three subtypes in TCGA cohort. [file Image_4.tif]

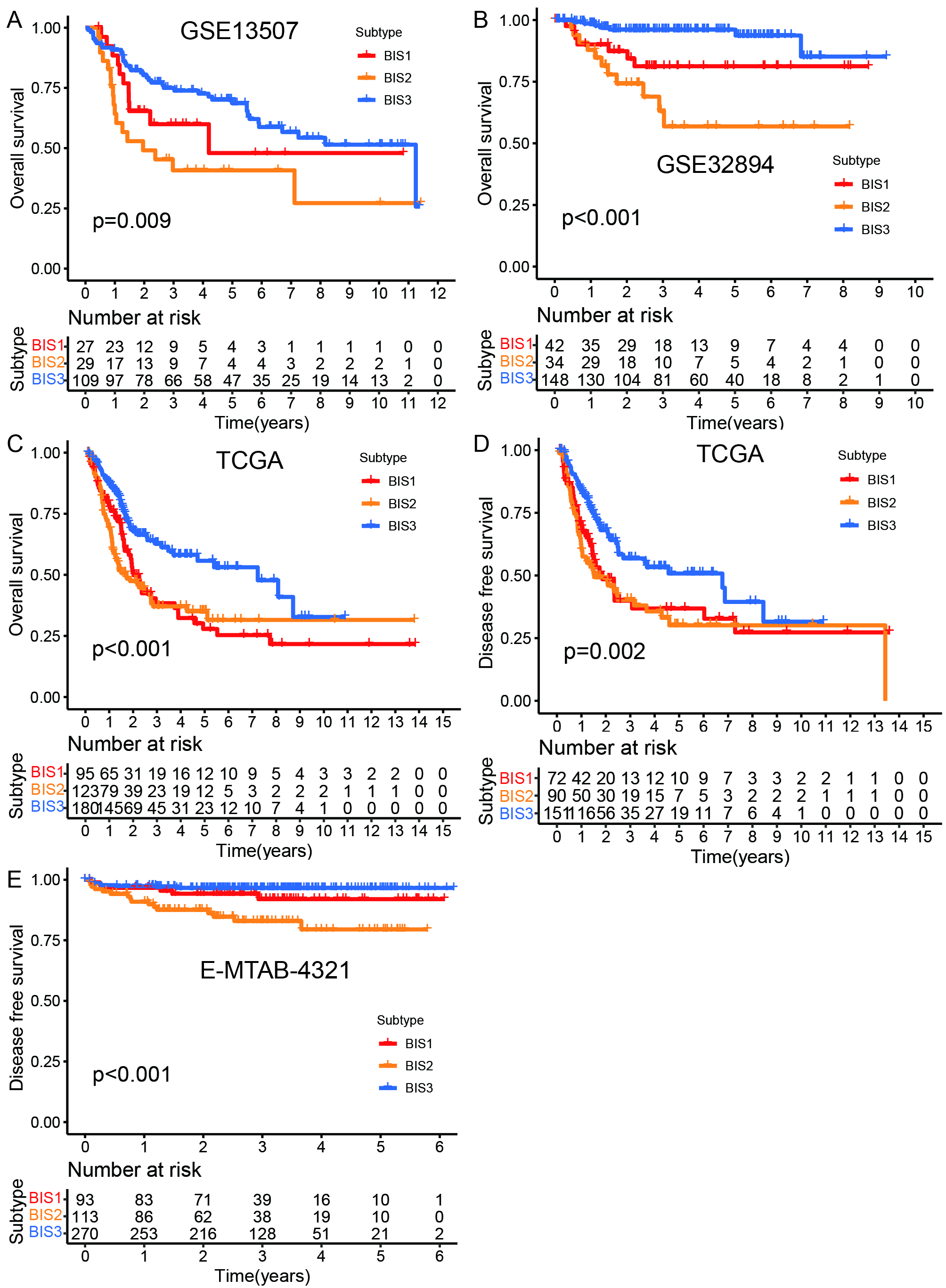

Supplement: Supplementary Figure 4 — The molecular characteristics of three subtypes. The keeg function enrichment analysis identifies the activated (A) and inhibited pathways (B) in each immune subtype. (C) The boxplot of ssGSEA score of signaling pathways between three immune clusters. (D) The boxplot of ssGSEA score of stem cell gene set between three immune clusters. [file Image_2.tif]

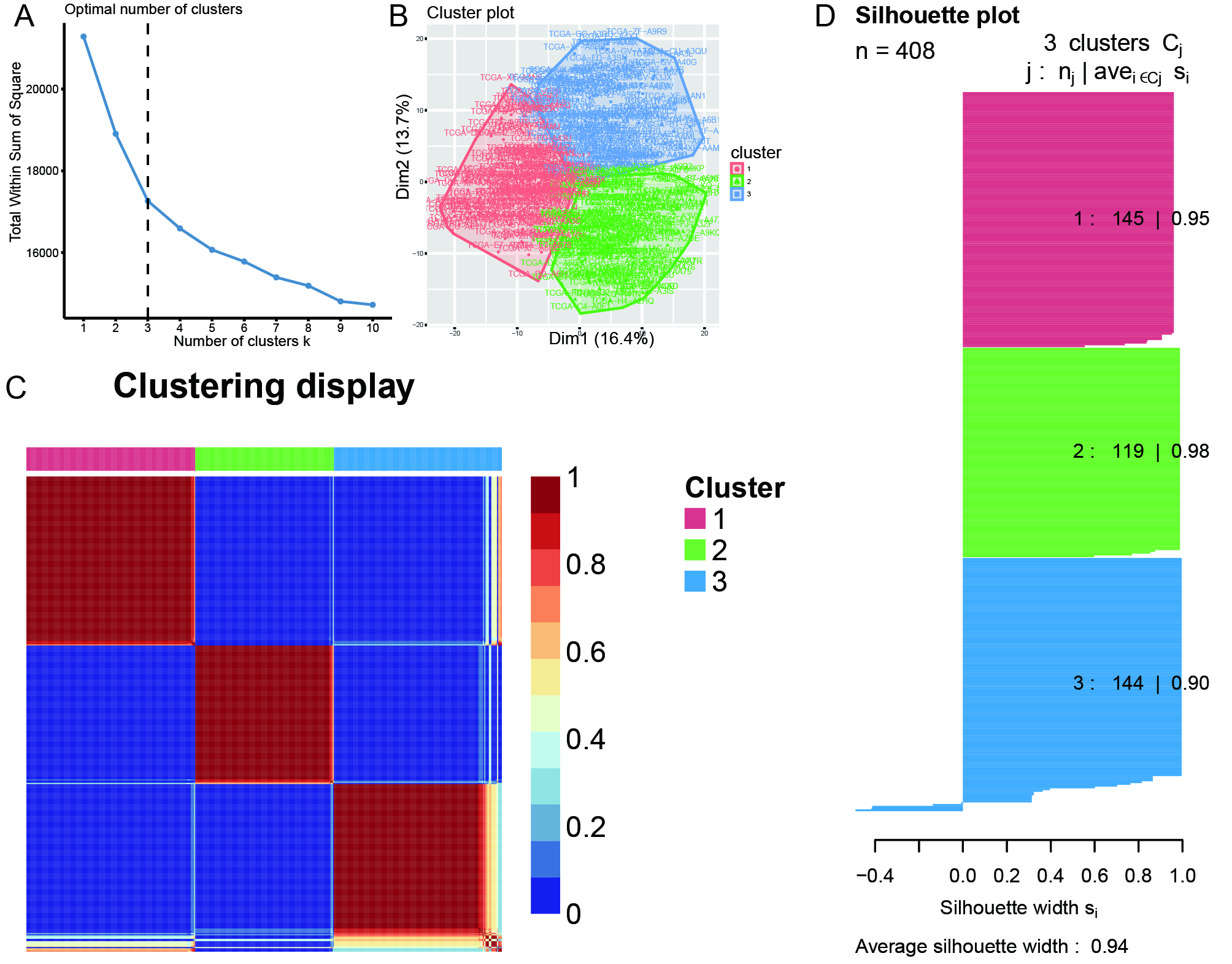

Supplement: Supplementary Figure 5 — Identification of methylation subtypes in BLCA. (A) The Total Whin Sum of Square of NMF analysis with different clusters number (k=1-10). (B) Stratification into three subtypes validated by PCA analysis in the Meta cohorts. (C) Heatmap representing the consensus matrix in the Meta cohorts. (D) The Silhouette index of each sample while BLCA samples were divided into three clusters by NMF analysis. [file Image_5.tif]

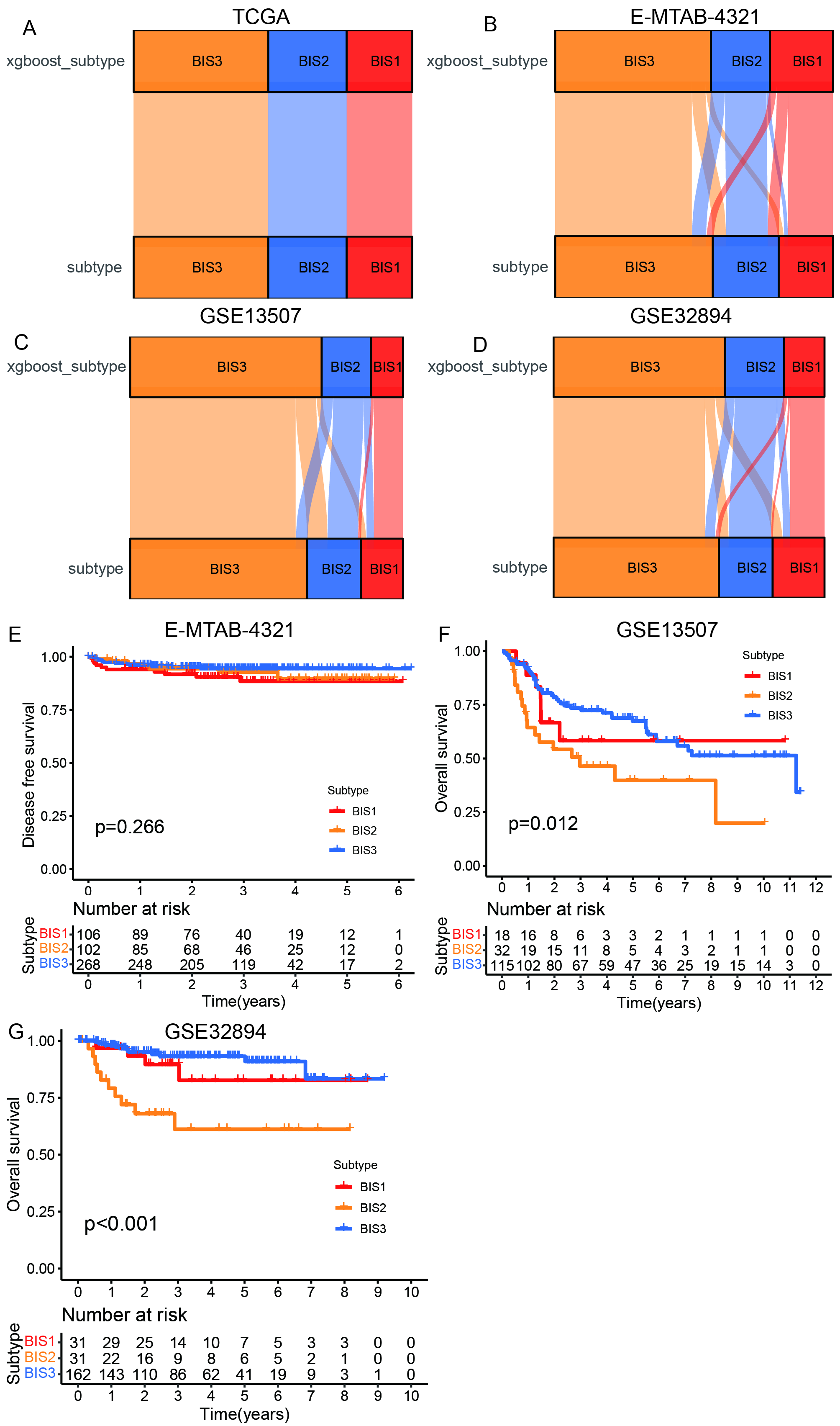

Supplement: Supplementary Figure 7 — The alluvial diagram of three subtypes predicted by XGBoost in TCGA (A), E-MTAB-4321 (B), GSE13507 (C), and GSE32894 (D) cohorts. Survival analysis between three subtypes predicted by XGBoost in E-MTAB-4321 (E), GSE13507 (F), and GSE32894 (G) cohorts. [file Image_7.tif]
